# Comparison of clinical and economic evaluation between selected generic and original febuxostat tablets in Chinese gout patients with hyperuricemia: A real-world multicenter retrospective study

**DOI:** 10.1097/MD.0000000000037081

**Published:** 2024-01-26

**Authors:** Xia Si, Lin Huang, Qingming Ding, Wei Zhang, Rui Zhao, Chao Ai, Zhuoling An, Gang Liu, Chunyan Zhang, Xue Zhong, Yufei Feng

**Affiliations:** a Department of Pharmacy, Peking University People’s Hospital, Beijing, China; b Department of Pharmacy, Beijing Tsinghua Changgung Hospital, Beijing, China; c Department of Pharmacy, Beijing Chao-Yang Hospital, Beijing, China.

**Keywords:** febuxostat, generic, gout, hyperuricemia, propensity score matching

## Abstract

Generic febuxostat tablets were listed in China’s third-round centralized drug procurement program. However, there are no sufficient data available on the use of febuxostat in a real-world setting. This study aimed to compare the efficacy, safety, and cost of selected generic febuxostat with original febuxostat in primary gout and hyperuricemia. Medical records at 3 tertiary hospitals from January 2014 to February 2022 were retrospectively analyzed. Propensity score matching was used to balance the distribution of baseline characteristics. The proportion of patients achieving target serum uric acid (SUA) levels at 12 weeks, the percent changes from baseline in SUA, adverse drug reactions, and the cost of febuxostat therapy were assessed. A total of 221 patients were recruited and 57 pairs of patients were 1:1 matched in the 2 groups. There was no statistically significant difference in the proportion of patients achieving a target SUA levels below 300 μmol/L, the percent changes of SUA decreased from baseline, and the incidence of adverse drug reactions between the 2 groups (all *P* > .05). The daily febuxostat cost in the generic group were significantly lower than that in original group (*P <* .05). Based on the results of this study, the clinical efficacy of selected generic febuxostat is comparable to that of original febuxostat for gout with hyperuricemia. No serious adverse reactions were reported in the 2 groups, and generic febuxostat is more economical than the original febuxostat.

## 1. Introduction

Serum uric acid (SUA) is the final metabolic product of purines in humans.^[1]^ In Chinese clinical practice and research, hyperuricemia is diagnosed when fasting SUA levels> 420 μmol/L after consumption of a normal purine diet on 2 separate days, regardless of gender.^[2,3]^ Hyperuricemia is a common disorder that affects patients of all ages and genders.^[4]^ Gout is an inflammatory arthritis caused by the deposition of monosodium urate crystals in the joints and soft tissues, the most important risk factor of which is hyperuricemia.^[5]^ Recently, a variety of studies have suggested that both gout and hyperuricemia are not only significantly associated with many cardiovascular and metabolic diseases,^[6]^ but also independent risk factors for premature mortality.^[7]^ Partly due to the unhealthy lifestyle and the aggravation of population aging, the prevalence and incidence of gout and hyperuricemia has been constantly increasing in China, with the younger generation being affected more.^[8,9]^ According to a recent survey from 31 provinces in mainland China, the weighted prevalence rates of hyperuricemia and gout in Chinese adults were 17.7% and 3.2%, respectively.^[9]^ The findings suggest hyperuricemia and gout have become major public health concerns in China, and urgent measures are needed to reduce the large burden of these conditions.

Pharmacological urate lowering therapy (ULT) is crucial for an optimal control of gout and hyperuricemia. However, urate-lowering agents are limited in number, with allopurinol and febuxostat being widely available, and probenecid, benzbromarone, and pegloticase available in some regions^.[10]^ Allopurinol remains the most commonly prescribed urate-lowering drug for hyperuricemia. Allopurinol is generally well tolerated, but it can cause severe or potentially life-threatening allopurinol hypersensitivity syndromes which require cessation of therapy.^[5]^ Notably, Han Chinese are high-risk populations for allopurinol-induced toxic epidermal necrolysis and Stevens-Johnson syndrome due to the common possession of the HLA-B*58:01 allele.^[11]^ Febuxostat, a novel non-purine selective xanthine oxidase, differs structurally from allopurinol and provides a potential alternative ULT in Chinese patients with allopurinol hypersensitivity syndrome.^[12]^

To reduce drug prices and improve the affordability of quality medicines, in November 2018, China launched a novel pooled procurement, the National Centralized Drug Procurement (NCDP) program.^[13]^ According to the NCDP policy, only generic drugs that had passed the Generic Consistency Evaluation and original branded drugs were eligible to be listed for procurement. Although generic drugs on the market are required to be bioequivalent to their corresponding original versions, however, their therapeutical equivalence may not necessarily be identical.^[14]^ A significant proportion of patients, pharmacists, and prescribers hold negative opinions about generic drugs, perceiving generic drugs as less effective, less safe and inferior in quality.^[15]^ Therefore, it is necessary to promote the clinical use monitoring and clinical comprehensive evaluation of NCDP-related drugs.^[16]^ Febuxostat is currently recommended as a first-line drug for patients with gout and hyperuricemia according to the Chinese clinical guidelines.^[3]^ In 2020, 3 generic febuxostat formulations (Fengdingning, Ruiyang and Youlitong) manufactured by different factories were listed in the third round of NCDP program; the bid-winning generic febuxostat tablet in Beijing was Fengdingning. However, patients with gout are considered at a high risk for cardiovascular disease,^[17]^ and data from clinical trial suggest that all-cause mortality and cardiovascular mortality were higher with febuxostat than with allopurinol in patients with gout and major cardiovascular coexisting conditions.^[18]^ It is necessary to evaluate the safety of febuxostat in a real world setting in China. To date, few studies have focused on the comparison between the efficacy, safety, and economy of generic and original febuxostat.

In this retrospective study, we therefore analyzed the clinical data from 3 tertiary hospitals in Beijing to explore the efficacy, safety, and economy of a selected generic febuxostat (Fengdingning) compared with original febuxostat (Feburic) for patients with gout and hyperuricemia in the real world.

## 2. Methods

### 2.1. Study design and participants

This was a multicenter retrospective cohort study. The electronic medical records of inpatients and outpatients diagnosed with gout and hyperuricemia who received original febuxostat (Feburic, Teijin Pharma Limited) or generic febuxostat (Fengdingning, HangZhou Zhuyangxin Pharmaceutical Co., Ltd) at Peking University People’s Hospital, Beijing Tsinghua Changgung Hospital and Beijing Chao-Yang Hospital from January 2014 to February 2022 were retrospectively collected, including clinical information (age, height, weight, history of gout, comorbidities, tophus, etc), prescriptions (including concomitant medications), and laboratory results (SUA, alanine aminotransferase, aspartate aminotransferase, etc). Gout was diagnosed by the treating doctor from clinical symptoms, physical examination findings, X-rays, and laboratory tests. Inclusion criteria: gout with hyperuricemia; aged between 18 and 70 years; received febuxostat tablets at a starting daily dose of 20 to 40 mg; baseline SUA ≥ 480 μmol/L or ≥ 420 μmol/L with one of the following conditions: gout flare frequency more than twice a year, tophus, chronic gouty arthritis, kidney stone, chronic kidney disease, hypertension, diabetes, dyslipidemia, stroke, ischemic heart disease, heart failure and the age of onset younger than 40 years old; complete clinical data. Exclusion criteria: acute gout attack; elevated SUA levels associated with organ transplant recipients, malignant diseases, Lesch-Nyhan syndrome, poisoning, other urate-lowering drugs or drugs that are known to affect SUA levels; treatment course of <12 weeks; missing clinical data. After screening, the patients were divided into 2 groups according to the drug regimen.

This study was approved by the Ethics Committee of Peking University People Hospital (ID: 2022PHb059-002), and the ethics approval has been circulated and endorsed by the other 2 hospital ethics committees before initiating the research. The written informed consent from patients was waived due to the retrospective nature of the study.

### 2.2. Treatment

According to package inserts, Feburic was recommended to start at a daily dose of 20 mg and Fengdingning at 20 to 40 mg, respectively. If SUA did not reach the target level, the dose of febuxostat was gradually increased to 80 mg daily. In clinical practice, the dose of Fengdingning and Feburic was initially prescribed based on the Chinese guidelines for the treatment of hyperuricemia and gout and the experience of clinicians, and then was adjusted clinically by patients’ tolerance to the drug. During treatment with febuxostat, other urate-lowering drugs were prohibited, while colchicine and/or nonsteroidal anti-inflammatory drugs were permitted if they experienced a gout flare.

### 2.3. Observation and outcome indicators

Parameters assessed: baseline and changes in SUA: the level of SUA at baseline and after 2, 4, 8, and 12 weeks of drug therapy; adverse drug reactions: relevant laboratory results (alanine aminotransferase, aspartate aminotransferase, etc), complaints (nausea, headache, etc) and concomitant medications (liver protecting drugs, nonsteroidal anti-inflammatory drugs, etc) were investigated to identify the most common and serious adverse drug reactions including abnormal liver function, gastrointestinal side effects, joint pain, headache, allergy and adverse cardiovascular effects,^[18,19]^ and the World Health Organization-Uppsala Monitoring Centre systems were applied for causality assessment^[20]^; costs: drug cost data derived from the hospital information system and the cost of febuxostat-related adverse events (including nursing, ward beds, laboratory tests, examinations, etc). All costs were represented in Chinese yuan.

Outcome indicators: The primary outcome was the proportion of patients achieving target SUA levels at 12 weeks. The secondary outcomes were percent changes in SUA levels at 2, 4, 8, and 12 weeks; adverse drug reactions, and costs.

### 2.4. Statistical analysis

Continuous data were presented as mean ± standard deviation if normally distributed or median with interquartile range if not normally distributed. Categorical data were described as numbers (n) and percentages (%). Student *t* test was used to test for group differences for continuous normally distributed variables with equal variance, and Mann–Whitney *U* test for non-normally distributed continuous variables or unequal variance was detected. Categorical variables were compared using chi-square test or Fisher exact test. A 1:1 propensity score matching (PSM) was applied to balance the baseline characteristics using the nearest-neighbor method with a caliper size of 0.02. Statistical analyses were carried out using SPSS statistical software (version 25.0). Two-tailed *P* values < .05 were considered statistically significant.

## 3. Results

### 3.1. Patient results

**Figure 1.**
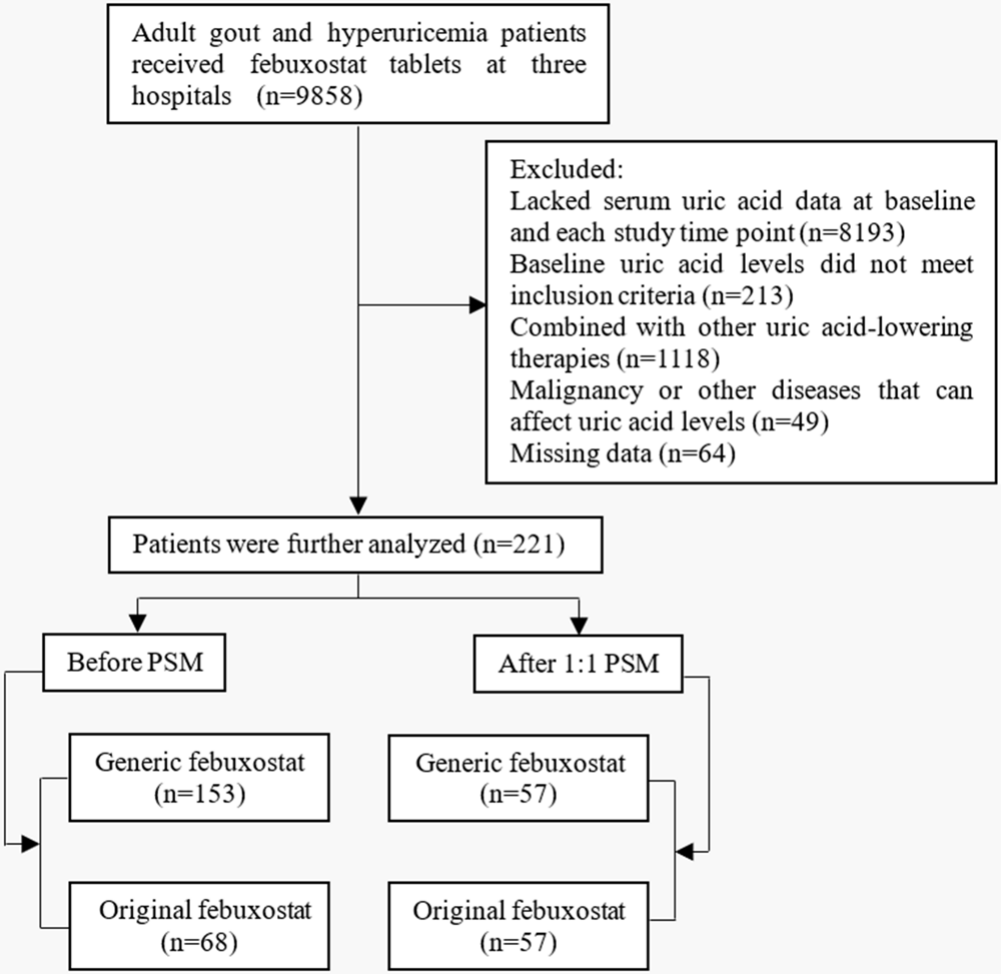
Flow chart of the study.

We identified 9858 gout and hyperuricemia patients who received febuxostat tablets at 3 hospitals from January 2014 to February 2022. According to the inclusion and exclusion criteria, 9637 patients were excluded. A total of 221 patients including 153 (69.23%) with generic febuxostat and 68 (30.77%) with original febuxostat were further assigned to the generic group (n = 57) and the original group (n = 57) by using PSM analysis with a 1:1 ratio (Fig. 1). Table 1 shows the characteristics of both groups before and after PSM. Before PSM, there were significant differences in age, onset age, chronic kidney disease, hypertension, dyslipidemia, and ischemic heart disease between the 2 groups (*P <* .05). After the performance of PSM, the baseline characteristics including sex, age, height, weight, body mass index, baseline SUA, and coexisting conditions were balanced between the 2 groups.

**Table 1:** Baseline characteristics before and after propensity score matching (PSM).

Characteristics	Before PSM			After PSM		
	Generic febuxostat (n = 153)	Original febuxostat (n = 68)	*P*	Generic febuxostat (n = 57)	Original febuxostat (n = 57)	*P*
Sex [n(%)]			0.063			.815
Male	104 (67.97)	53 (77.94)		46 (80.70)	45 (78.95)	
Female	49 (32.03)	15 (22.06)		11 (19.30)	12 (21.05)	
Age (yr), mean ± SD	53.78 ± 15.29	46.09 ± 15.15	0.001	47.82 ± 15.64	47.65 ± 15.00	.951
Height (cm), mean ± SD	167.91 ± 8.40	170.49 ± 8.40	0.068	170.24 ± 7.29	170.53 ± 8.76	.873
Weight (kg), mean ± SD	70.13 ± 12.41	69.89 ± 13.25	0.909	72.95 ± 11.80	69.47 ± 13.11	.209
BMI (kg/m^2^), mean ± SD	24.82 ± 3.73	24.01 ± 4.09	0.213	25.12 ± 3.53	23.89 ± 4.13	.156
Gout feature						
Baseline SUA (μmol/L), mean ± SD	582.25 ± 106.20	554.88 ± 87.05	0.064	578.86 ± 102.97	550.35 ± 88.02	.115
Onset age ˂40 yr [n(%)]	33 (21.57)	28 (41.18)	0.003	22 (38.60)	21 (36.84)	.847
˃2 gout flares/yr [n(%)]	1 (0.65)	1 (1.47)	0.522	1 (1.75)	0 (0)	1.000
coexisting conditions [n(%)]						
Tophus	2 (1.31)	3 (4.41)	0.171	0 (0)	2 (3.51)	.496
Chronic gouty arthritis	18 (11.76)	11 (16.18)	0.370	5 (8.77)	7 (12.28)	.542
Kidney stone	7 (4.58)	4 (5.88)	0.938	4 (7.02)	4 (7.02)	1.000
Chronic kidney disease	145 (94.77)	57 (83.82)	0.007	50 (87.72)	49 (85.96)	.782
Hypertension	130 (84.97)	50 (73.53)	0.043	42 (73.68)	44 (77.19)	.663
Diabetes	65 (42.48)	28 (41.18)	0.856	23 (40.35)	23 (40.35)	1.000
Dyslipidemia	78 (50.98)	48 (70.59)	0.007	38 (66.67)	38 (66.67)	1.000
Stroke	9 (5.88)	5 (7.35)	0.908	4 (7.02)	4 (7.02%)	1.000
Ischemic heart disease	15 (9.80)	16 (23.53)	0.007	11 (19.30)	10 (17.54)	.809
Heart failure	7 (4.58)	3 (4.41%)	1.000	2 (3.51)	2 (3.51)	1.000

BMI = body mass index, SD = standard deviation, SUA = serum uric acid.

### 3.2. Achievement of the target SUA level

Chinese guidelines of hyperuricemia and gout recommend that target SUA levels of ULT should be maintained at <360 μmol/L or <300 μmol/L in those with severe gout.^[3]^ As presented in Table 1, all of the 114 propensity-matched patients suffered from at least one of the following conditions, such as chronic kidney disease, hypertension, and diabetes. Thus, the target of ULT was set as the endpoint SUA level <300 μmol/L and the number and proportions of patients who achieved that target were calculated. After 12 weeks of treatment, 52.63% (30 of 57 patients) in the generic febuxostat group and 57.89% (33 of 57 patients) in the original febuxostat group achieved an SUA level <300 μmol/L (Table 2). There was no significant difference in the proportion of patients with target SUA at 12 weeks after initiation of ULT between the 2 groups (*P* = .572).

**Table 2:** Achievement of the target SUA at 12 wk after treatment in a propensity score-matched cohort.

Treatment	n	Target [n(%)]	Not on target [n(%)]	χ^2^	*P*
Generic febuxostat	57	30 (52.63)	27 (47.37)	0.319	.572
Original febuxostat	57	33 (57.89)	24 (42.11)		

SUA = serum uric acid.

### 3.3. Percentage change in SUA levels

Table 3 shows the percentage changes calculated from baseline SUA levels after 2, 4, 8, and 12 weeks of treatment in the generic and original febuxostat groups. The reduction of SUA levels in the original febuxostat group were larger than in the generic febuxostat group at each time point, while no significant difference was observed between the 2 groups (all *P* > .05).

**Table 3:** Percentage changes from baseline SUA levels at 2, 4, 8, and 12 wk after treatment in the propensity score-matched cohort.

Duration (wk)	Generic febuxostat (%) (n = 57)	Original febuxostat (%) (n = 57)	*t*	*P*
2	−33.10 ± 18.37	−38.62 ± 17.66	−1.634	.849
4	−38.72 ± 19.01	−42.82 ± 15.26	−1.269	.224
8	−38.97 ± 16.75	−42.41 ± 13.11	−1.223	.291
12	−41.67 ± 14.17	−43.02 ± 15.03	−0.496	.500

SUA = serum uric acid.

### 3.4. Adverse drug reactions

Safety was assessed in 221 patients (153 in the generic febuxostat group and 68 in the original febuxostat group) before PSM and 114 patients (57 in the generic febuxostat group and 57 in the original febuxostat group) after PSM. No deaths occurred in this study. The most commonly reported adverse effects were elevated alanine transaminase and aspartate transaminase levels, with no significant difference in alanine transaminase and aspartate transaminase levels before and after PSM (all *P >* .05). One patient experienced joint pain in the generic febuxostat group before PSM. No other adverse reactions related to generic or original febuxostat tablets were recorded in both groups within 12 weeks (Table 4).

**Table 4:** Adverse events occurring during treatment.

Adverse event	Before PSM			After PSM		
	Generic febuxostat (n = 153)	Original febuxostat (n = 68)	*P*	Generic febuxostat (n = 57)	Original febuxostat (n = 57)	*P*
Joint pain [n(%)]	1 (0.65)	0	–	0	0	–
Elevated alanine transaminase [n(%)]	28 (18.30)	19 (27.94)	.106	11 (19.30)	15 (26.32)	.372
Elevated aspartate transaminase [n(%)]	6 (3.92)	3 (4.41)	1.000	2 (3.51)	2 (3.51)	1.000

PSM = propensity score matching.

### 3.5. Cost of febuxostat

In this study, both generic and original tablets were administered orally with similar efficacy and safety for treating gout and hyperuricemia. The difference in treatment cost between the 2 groups mainly came from unit prices and daily doses of febuxostat. The mean daily doses and costs of febuxostat tablets were calculated in patients who achieved the target SUA level at week 12 of treatment. 30 patients in the generic febuxostat group and 33 patients in the original febuxostat group were included in the economic evaluation (Table 5). During the 12-week study period, the mean daily dose of generic febuxostat was significantly higher than that in the original group (*P <* .05). However, due to the low price of bidding generic febuxostat, the mean daily cost of generic febuxostat was significantly lower than that in the in the original group (*P <* .05).

**Table 5:** Mean daily doses and costs of febuxostat for patients who achieved the target SUA level in the propensity score-matched cohort.

Treatment	n	Unit price (CNY/mg)	Mean daily dose (mg)	*t*	*P*	Mean daily cost (CNY)	*t*	*P*
Generic febuxostat	30	0.04	36.10 ± 11.75	−5.236	<.001	1.44 ± 0.47	12.76	<.001
Original febuxostat	33	0.21	23.40 ± 7.15			5.02 ± 1.53		

CNY = Chinese yuan, SUA = serum uric acid.

## 4. Discussion

In the present multicenter, retrospective observational study, we demonstrated that selected generic febuxostat had comparable clinical effectiveness and less medical costs compared with the original formulation. As the first study to compare the clinical outcomes and medical costs of generic and original febuxostat tablets in China, we provide critical evidence for low price generic substitution of febuxostat to reduce medical burden of patients and government.

A treat-to-target management strategy is strongly recommended for all patients receiving ULT.^[21]^ The goal of ULT is to achieve a target of SUA < 360 μmol/L in all gout patients and or <300 μmol/L for gout patients with greater disease severity and urate burden by the 2019 Chinese guidelines for the diagnosis and management of gout and hyperuricemia. A total of 114 patients enrolled after PSM had at least one comorbid condition, partly because patients admitted to the 3 included top tertiary hospitals in China were relatively severe and febuxostat was widely prescribed in patients with mild to moderate kidney or liver impairment for requiring no dose adjustment. Different from previous studies,^[22,23]^ the target of ULT was set as the endpoint SUA level <300 μmol/L in this study. The results showed that, in a real-world setting, generic febuxostat had similar urate-lowering efficacy at 2, 4, 8, and 12 weeks compared with original febuxostat. The proportions of patients who achieved the serum urate target of below 300 μmol/L at 12 weeks were 52.63% in the generic group and 57.89% in the original group (*P >* .05).

The findings from our study suggested good safety profiles of both generic and original febuxostat tablets, despite patients with complex comorbidities. The most frequent adverse events were mild to moderate elevations of liver aminotransferases (alanine transaminase more than aspartate transaminase), which was consistent with previous reports.^[22,24]^ There were no skin reactions, gastrointestinal reactions, or major cardiovascular adverse effects induced by febuxostat in either group. As the data were collected through electronic medical records rather than through self-report, the incidence of adverse effects such as gout flares may be underestimated.

Our study found comparable clinical outcomes of generic and original febuxostat tablets, thus the economic differences between the 2 groups were mainly from unit prices and daily doses of febuxostat tablets, regardless of the additional costs of individual adverse reactions or other factors. The label recommendation for the starting dose of Fengdingning was 40 mg daily when it was on the market in 2013. Abrupt decreases in SUA level might precipitate gout attacks, then the initial dose of Fengdingning was revised to 20 mg daily in 2018, the same as the starting dose of Feburic. Thus, the most frequently prescribed initial daily dose in this study cohort was 40 mg for Fengdingning and 20 mg for Feburic. After calculation, the daily dose of Fengdingning was significantly higher than that of Feburic (*P <* .05). However, due to the low bid price of Fengdingning, the daily cost of Fengdingning was significantly lower than that of Feburic (*P <* .05) in the treatment of gout and hyperuricemia. It is expected that the daily dose and cost of generic febuxostat will be further reduced according to the latest revision of the instructions.

Our study had a few strengths. To the best of our knowledge, this was the first study using patient records to compare the clinical effects and safety between policy-related generic and original febuxostat tablets, which can provide critical evidence for public health policy and practice in the treatment of gout with hyperuricemia and act as a reference for other countries with situation similar to China. Besides, we applied a PSM study design to balance potential confounding in observational studies and improve the strength of findings.

Our study also had several limitations. First, the study was a retrospective analysis which might be subjected to selection bias and other potential confounding factors. Second, diet, exercise, and other medical conditions can also affect the SUA level, we could not verify the uric acid lowering effects were entirely attributed to febuxostat. Third, the number of patients included in the present study was relatively small. We checked the records of all 9858 patients and found that about 9637 (97.76%) of them were excluded from the analysis. The main reason for exclusion was a lack of SUA data (83.11%), suggesting poor compliance or inadequate medication or mild illness. Loss to follow-up is a common phenomenon and challenge in retrospective studies. In the future, rigorously designed cohort studies or RCTs will be required to further confirm the findings of the current research. Fourth, all included patients suffered from severe gout manifestations or other comorbidities, and clinical efficacy was evaluated based on a strict target SUA levels of <300 μmol/L. Additional study was needed to assess the clinical effects of febuxostat on patients without complications.

## 5. Conclusions

Generic febuxostat showed comparable urate-lowering efficacy and lower medical costs compared with original febuxostat in the treatment of Chinese gout patients with hyperuricemia within 12 weeks. More data are needed to evaluate the long-term efficacy, safety, and economy of generic versus original febuxostat tablets for gout and hyperuricemia. The results of our study may provide significant evidence and insights for better hyperuricemia management in China and other countries with situation similar to China. These could also help increase patients and healthcare providers’ confidence and adherence to generic febuxostat tablets and ultimately improve SUA control rate.

## Acknowledgments

The authors thank Pingjin Zhang, Jun Liu, Jiajie Luan and Yanqing Song for their help in this study.

## Author contributions

**Conceptualization:** Xia Si, Lin Huang, Yufei Feng.

**Data curation:** Wei Zhang, Rui Zhao, Chunyan Zhang, Xue Zhong.

**Formal analysis:** Xia Si.

**Funding acquisition:** Yufei Feng.

**Methodology:** Qingming Ding.

**Software:** Gang Liu.

**Supervision:** Wei Zhang, Rui Zhao, Chao Ai, Zhuoling An.

**Writing – original draft:** Xia Si.

**Writing – review & editing:** Lin Huang, Yufei Feng.
